# Temporal trends in radiation-induced optic neuropathy: a meta-analysis

**DOI:** 10.1016/j.ctro.2026.101327

**Published:** 2026-10-18

**Authors:** Juliette Thariat, Thao-Nguyen Pham, Thomas Sochacki, Florent de Vathaire, Helen A. Shih, Jean-Claude Quintyn

**Affiliations:** a Department of Radiation Oncology, Centre François Baclesse, Caen, Normandy, France; b Laboratoire de physique corpusculaire UMR6534 IN2P3/ENSICAEN, Caen-Normandy University, France; c CESP, INSERM U1018, Cancer and Radiation team, Gustave Roussy Institute, Villejuif, France; d Department of Radiation Oncology, Massachusetts General Hospital/Mass General Brigham, Harvard Medical School, Boston, MA, United States of America; e Department of Ophthalmology, University Hospital, Caen, Normandy, France

**Keywords:** Tumor, Radiotherapy, Brachytherapy, Proton therapy, IMRT, Toxicity, Optic neuropathy, Meta-analysis

## Abstract

**Background:**

Radiation-induced optic neuropathy (RION) is a rare but disabling toxicity. This systematic review and meta-analysis aimed to evaluate temporal trends in the incidence of RION according to advances in radiotherapy techniques and fractionation schemes over time.

**Methods:**

A systematic search identified 1994–2025 studies reporting optic neuropathy in patients after radiotherapy (hereby defined as RION). Data on RION incidence, diagnostic criteria, ocular or extraocular tumor, radiotherapy modality, conventional fractionation or hypofractionation, and modeling strategies were extracted. Random effects meta-analysis was performed to estimate pooled incidence rates and assess heterogeneity.

**Findings:**

The pooled RION incidence for ocular tumors was 17.0% (95%CI:12.1–23.5%; range 0.0–81.7%; 1.3–11.0-year follow-up) (brachytherapy studies = 33, proton therapy = 3). Risk factors included tumor proximity to the optic nerves, dose exceeding 50Gy in 2Gy-equivalent dose (EQ.D2), tumor size, and patient comorbidities. The pooled RION incidence for extraocular tumors was 4.7% for conventionally fractionated (39 studies, 5.1-year follow-up) and 2.1% for hypofractionated radiotherapy (28 studies, 3.4-year follow-up). Head-and-neck tumors were significantly associated with a greater incidence of RION (estimate = 0.7185, *p* = 0.0076). Among extraocular tumors, only central nervous system (CNS) tumors underwent hypofractionated radiotherapy. Using meta-regression, fractionation, fraction size, and EQ.D2 did not significantly influence RION incidence in CNS tumors. Median follow-up time varied widely between studies. Meta-regression using recruitment period as a covariate did not identify a statistically significant temporal trend in RION incidence (*p* = 0.183). Dedicated meta-regressions on publication year, with and without outlying studies, likewise showed no significant temporal trend in intraocular (odds ratio [OR] per decade 0.87, *p* = 0.51), conventionally fractionated extraocular (OR 0.94, *p* = 0.74) or CNS tumors (OR 0.90, *p* = 0.46).

**Interpretation:**

RION incidence varies with tumor location, radiotherapy modality, and fractionation. Although strongly dose-dependent, RION is also influenced by pre-existing optic pathway damage, ocular comorbidities (e.g., glaucoma, diabetes), and multimodal treatments. These findings highlight the limits of dosimetric optimization alone and support a shift toward biologically driven strategies. Therapeutic development or repurposing requires a deeper mechanistic understanding of RION, for which high-resolution ophthalmological imaging offers a unique in vivo approach to identify actionable targets.

## Highlights

• Radiation-induced optic neuropathy (RION) remains a rare but debilitating RT toxicity.
• RION incidence varies markedly across tumor sites.
• Optic pathway dose and tumor proximity are the dominant risk determinants.
• RION incidence has remained stable across radiotherapy modalities in the last 2 decades.
• Reduction of RION incidence may arise from better understanding of mechanisms and specific therapies.

## Graphical abstract

**Figure:**
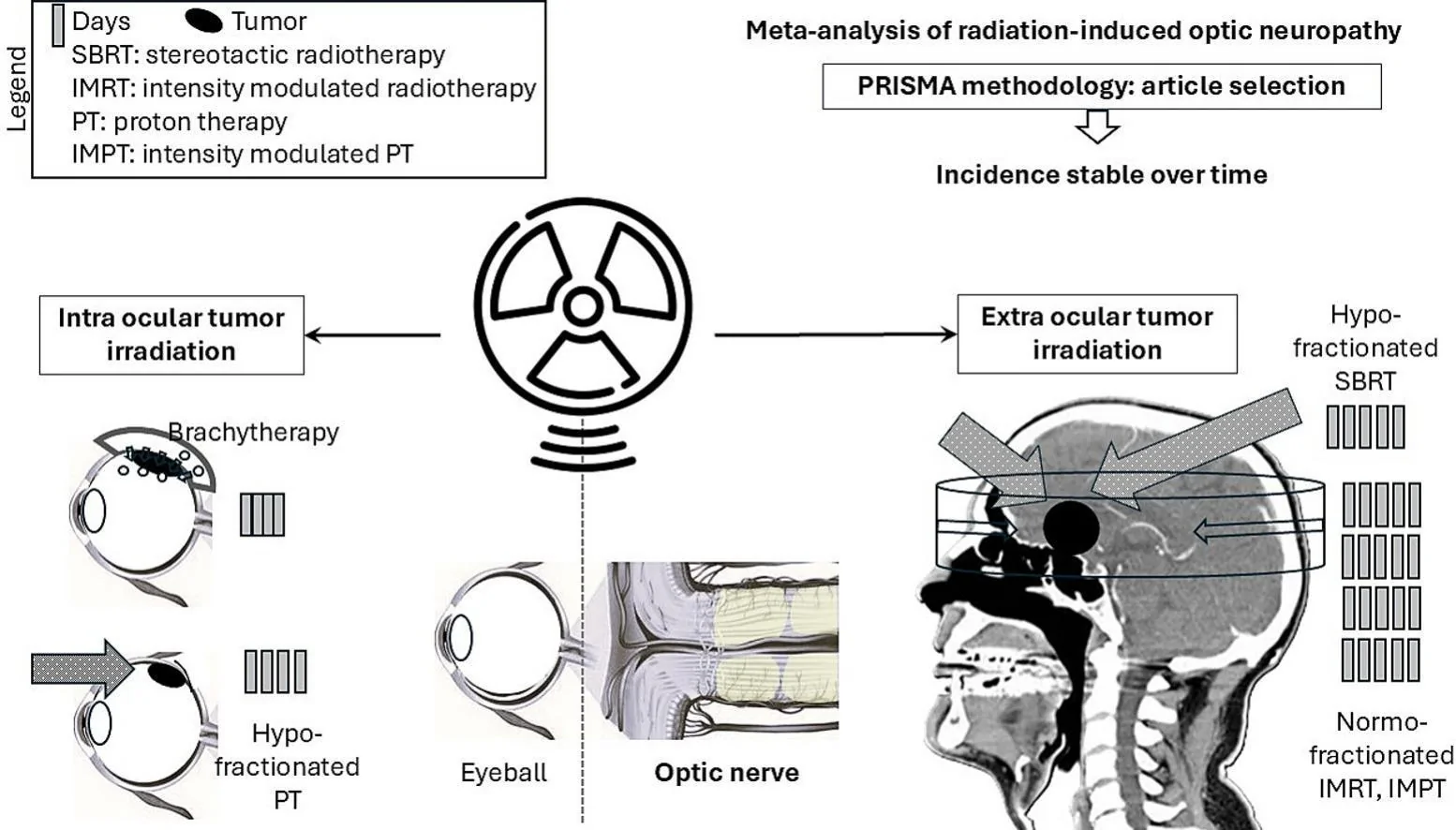

**Research in context**

**Evidence before this study:** Radiation-induced optic neuropathy (RION) is an uncommon but functionally disabling complication of radiotherapy in the context of central nervous system (CNS), head and neck, skull base close to the visual pathways, and intraocular tumors. Historical data from Leber et al. (1998) and Parsons et al. (1994) established foundational dose–response relationships in conventionally fractionated (<2.5Gy fractions) radiotherapy, with doses exceeding 50–55 Gy to the optic nerve or chiasm significantly increasing RION risk. Subsequent studies introduced biological models, such as the linear-quadratic model and normal tissue complication probability (NTCP) frameworks, but these remained limited in their applicability to hypofractionated (≥2.5Gy fractions) or ultra-hypofractionated (≥5Gy fractions) regimens due to uncertain α/β ratios and poor clinical validation.

With increasing use of high-precision radiotherapy, such as intensity-modulated radiotherapy, proton therapy, and stereotactic techniques, new uncertainties have emerged regarding RION risk, especially with altered fractionation schedules, different dose distributions and potentially different physical/radiobiological mechanisms. However, existing evidence has been fragmented. Most prior reports are retrospective, single-center studies with heterogeneously defined RION diagnoses and limited external validity. No prior meta-analysis has systematically quantified RION incidence across different tumor locations or radiotherapy modalities.

**Added value of this study:** This study provides the most comprehensive synthesis of RION risk to date, including 103 studies. We present pooled RION incidence estimates stratified by tumor site, radiotherapy modality and fractionation. Pooled RION incidence was 17.0% in intraocular tumors, with highest rates in juxtapapillary tumors, and up to 4.7% in extraocular CNS/head-and-neck tumors. These clinically relevant rates were associated with risk factors, such as radiation doses exceeding 50 Gy, proximity to the optic nerve, and larger tumor volumes. The analysis also revealed inconsistencies in diagnostic criteria and underreporting in hypofractionated cohorts.

**Implications of all the available evidence:** This study reinforces that RION remains a disabling and clinically-relevant risk across a wide range of tumor types and radiotherapy techniques. Although modern techniques have improved target conformality and reduced off-target exposure, RION incidence remains substantial in intraocular tumors and CNS/head-and-neck tumors close to the optic nerve. Current NTCP models based on conventional fractionation may underestimate risk in clinical contexts where dose per fraction exceeds 6 Gy. Our findings highlight the need for prospective studies using standardized diagnostic criteria and patient-level dosimetric data to improve model reliability.

## 1 Introduction

Radiation-induced damage to the visual pathway is a debilitating complication of radiotherapy of tumors of the brain, skull base, orbit, or paranasal sinuses, as well as intraocular tumors [1]. Radiation-induced optic neuropathy (RION) can lead to vision loss because it is often detected at a late stage where it is irreversible. RION is considered a subset of delayed radiation-induced necrosis [2], but its exact mechanisms, with potential damage to axons, endothelial cells or myelin, are not fully understood. Corrective treatments for RION are mostly ineffective, making RION a significant concern in patients receiving radiotherapy [3]. Advances in high-resolution and multimodal imaging, including contrast-enhanced magnetic resonance imaging (MRI), optical coherence tomography (OCT), and OCT angiography (OCT-A), may enable earlier detection and longitudinal characterization of radiation-induced structural and microvascular changes, potentially identifying a subclinical window before overt visual loss [4], [5], [6]. RION has been reported after all types of radiotherapy, including conventionally fractionated and hypofractionated radiotherapy. Intraocular tumors, such as uveal melanomas, are treated with high-precision high-dose small beam irradiation while giving as low-as-reasonably-achievable dose to the ipsilateral optic nerve. Recent advances in radiotherapy techniques, and combination with steroids or Vascular Endothelial Growth Factor inhibitors, have been introduced to optimize radiotherapy precision and reduce visual toxicity. Extraocular tumors are often treated with large radiation fields and conventional fraction size or smaller fields and larger fraction size. Unfortunately, effective therapeutic strategies for RION remain limited in both ocular and extraocular CNS/head-and-neck tumor settings [7], [8], [9].

On the basis of historical models based on dose parameters, radiation dose constraints are systematically applied in radiotherapy to the optic nerves and chiasm, with the maximum dose set to approximately 54 Gy EQ.D2 [10] [11]. In certain situations, compromises may be needed to efficiently treat a tumor abutting one optic nerve; it is not uncommon to have to prioritize the protection of one eye while accepting the potential loss of the other. In contrast, it is very uncommon to take a risk of bilateral damage by irradiating both nerves or the chiasm above their tolerance dose. Optic nerve tolerance is also influenced by fraction size. The optic nerve is generally considered a late-responding tissue with a low α/β ratio, commonly estimated at approximately 2–3 Gy, and is therefore sensitive to increases in dose per fraction. With increasing use of hypofractionated radiotherapy, refinements of RION NTCP models, defined for conventionally fractionated radiotherapy, are needed 12. Additional challenges are posed by statistical uncertainties in RION definitions and endpoints, data compilation, inaccuracies in α/β values, the applicability of the linear-quadratic model (which is known to be inadequate above 6 Gy per fraction), and the lack of data at both low- and high-dose extremes.

The present systematic review and meta-analysis aimed to evaluate the incidence of RION over the years across intraocular and extraocular tumors, examine associated clinical and treatment-related risk factors, and explore differences related to radiotherapy modalities.

## 2 Methods

### 2.1 Search strategy and selection criteria

#### 2.1.1 Literature search

This systematic review was conducted following the Preferred Reporting Items for Systematic Reviews and Meta-Analyses (PRISMA) guidelines. A comprehensive search of the PubMed database was performed via the following MeSH terms: (radiotherapy [Title/Abstract]) AND (optic neuropathy [Title/Abstract]). The literature search included terms related to radiotherapy and radiation-induced optic neuropathy, supplemented by screening of reference lists of eligible articles. Because the primary search was restricted to PubMed, incomplete capture of studies indexed exclusively in other databases cannot be excluded and is acknowledged as a limitation. Clinical and modeling studies of intraocular and extraocular tumors were selected when providing information on RION. Studies involving any form of radiotherapy (including brachytherapy, intensity-modulated radiation therapy (IMRT) and proton beam therapy (PBT), stereotactic irradiation) were considered. Only articles published in English up to January 2025 were included. Eligible clinical studies had to include a minimum of 20 participants and provide data on RION incidence, associated risk factors, NTCP modeling, dose constraints, and radiotherapy techniques. Case reports and review articles were excluded.

All identified records were screened for duplicates. When updated articles on the same study population were found, the versions with larger sample sizes were retained. Two independent reviewers assessed article eligibility in a multistep process, beginning with titles, followed by abstracts, and finally, a full-text review. Inter-reviewer agreement was substantial at the abstract and final-eligibility stages (Cohen's κ = 0.68 and 0.65, respectively); disagreements were resolved by consensus. Studies were excluded if they lacked adequate data on RION assessment, did not report radiation dose constraints, or focused solely on non-radiotherapy-induced optic neuropathy or pre-radiotherapy visual recovery. The reference lists of the selected articles were reviewed to identify any additional studies. The overall search strategy is illustrated in the PRISMA flowchart (Fig. 1). Risks of bias were evaluated using the Newcastle-Ottawa Scale (NOS) (Supplementary material). Studies scoring ≥8/9 were considered of high quality, and the NOS score was examined as a moderator in meta-regression.

**Fig. 1:**
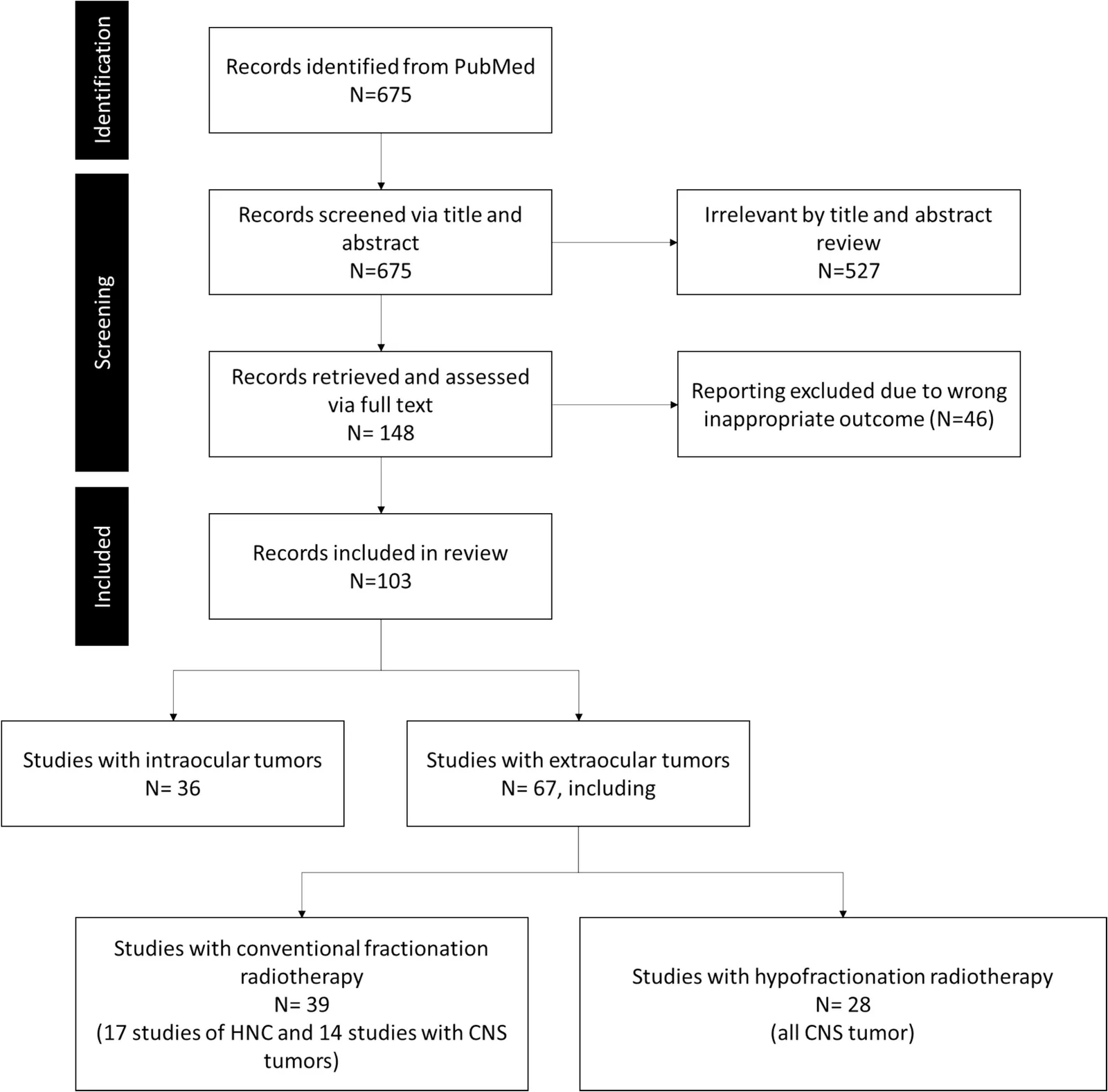
Radiation-induced Optic Neuropathy Reporting Items for Systematic Reviews and Meta-Analyses (PRISMA) flow diagram representing the approach to identification, screening, eligibility, and inclusion of articles. Legend: Risk of bias of the included studies was assessed with the Newcastle–Ottawa Scale (Supplementary Material).

### 2.2 Data analysis

A meta-analysis was conducted to synthesize reported proportions of RION across eligible studies. The definition of RION, radiation parameters, technique and fractionation were collected. For intraocular tumors, brachytherapy and proton therapy effects were analyzed with particular attention given to how each technique influences RION incidence. When fractionation and dose to the organs at risk were compared under different radiotherapy techniques, the equivalent dose in 2 Gy fractions (EQ.D2) was used. Conventional fractionation was defined as 1.8–2.5 Gy per fraction, hypofractionation as delivery of >2.5 Gy per fraction, ultrahypofractionation as ≥5 Gy per fraction. For consistency, the terms “conventional fractionation” and “conventionally fractionated” are used throughout the manuscript and Supplementary Material. For proton therapy, a relative biological effectiveness (RBE) of 1.1 was used to convert the physical dose to a photon-equivalent dose, which is consistent with clinical practice and ICRU Report 78 13. Reported maximum optic-structure dose (Dmax) was extracted as defined in each source study. Because the sampled volume was frequently unspecified and definitions varied across treatment eras, reported Dmax values were not assumed to be equivalent to D0.03 cc.

Outliers were identified using the interquartile range (IQR) method based on the reported incidence rates of RION across studies within each subgroup. Specifically, a study was considered an outlier if its reported RION incidence exceeded 1.5 times the IQR above the third quartile (Q3) or below the first quartile (Q1) 14. The statistical analysis employed a random-effects model to accommodate anticipated heterogeneity. Proportions were transformed via the logit function to stabilize variances prior to pooling. Each study's estimate was weighted via the inverse variance method, which gives greater weight to more precise estimates. Between-study heterogeneity was assessed via the restricted maximum likelihood (REML) approach and quantified through Cochran's Q statistic, the I^2^ index, and the H statistic. Confidence intervals (CI) for between-study variance (τ^2^) and its standard deviation (τ) were calculated via the Q-profile method. Clopper-Pearson exact methods were used to derive individual study confidence intervals. Meta-regression analyses were conducted to examine the effects of study size, follow-up duration, radiation technique, and patient factors on RION incidence. To assess temporal trends, publication year and, alternatively, the midpoint of the patient recruitment period were entered as continuous covariates in random-effects meta-regressions (logit scale; results expressed as odds ratios [OR] per decade) within each subgroup, both unadjusted and adjusted for follow-up duration, RION definition, tumor type, fractionation and dose where applicable; pooled incidence was also estimated by publication era (<2000, 2000–2009, ≥2010). These meta-regressions used the Knapp–Hartung adjustment, and the robustness of the exploratory ascertainment-adjusted model was assessed with a permutation test (2000 iterations). Key pooled estimates and meta-regressions were computed both with and without IQR-defined outliers. Additional sensitivity analyses used a binomial-normal generalized linear mixed model (GLMM), reported 95% prediction intervals, and explored study quality (NOS score) as a moderator. All analyses were performed via validated meta-analysis software in accordance with current best practices. Meta-regression and meta-analysis were performed via the meta (version 8.0.1) and metafor (version 4.6.0) packages in RStudio (version 2024.09.1).

## 3 Results

An initial search identified 675 records, which were screened by title and abstract. A total of 148 studies underwent full-text review. After applying the inclusion and exclusion criteria, 103 studies were included in the final analysis (Fig. 1). Among these, 36 studies examined RION in intraocular tumors, and 67 in extraocular tumors. Within the extraocular group, 39 studies used conventionally fractionated radiotherapy techniques (including 17 studies of head and neck cancer and 14 studies of CNS tumors), and 28 employed hypofractionated radiotherapy (all being CNS tumors).

### 3.1 RION in intraocular tumors

The 36 intraocular tumor studies published between 1994 and 2025 reported RION incidence rates ranging from 0% to 81.72%, with follow-up durations ranging from 1.3 to 11 years (Table 1). The majority (91.67%, 33/36) used brachytherapy plaques of ruthenium-106, iodine-125, or palladium-103, positioned under a short general anesthesia and continuously applied for several days before removal. The remaining three studies used hypofractionated PBT delivered over several days in fractions exceeding 10 Gy.

**Table 1:** Radiation-induced Optic Neuropathy Incidence in Series of Intraocular Tumors.

Studies	Study size	Follow-up period (years)	Tumor Type	Radiotherapy type	RION (%)	RION Diagnostic Methods
[15]	608	11.00	Uveal Melanomas	Brachytherapy	18.75	Undefined
[16]	186	3.83	Retinoblastomas	Brachytherapy	15.59	Undefined
[17]	109	2.08	Uveal Melanomas	Brachytherapy	44.04	Imaging
[18]	56	5.50	Uveal Melanomas	Brachytherapy	12.50	Mixture
[19]	225	2.80	Uveal Melanomas	Brachytherapy	20.44	Imaging
[20]	258	2.79	Uveal Melanomas	Brachytherapy	21.71	Mixture
[21]	219	4.71	Uveal Melanomas	Brachytherapy	10.96	Imaging
[22]	258	8.50	Uveal Melanomas	Brachytherapy	3.10	Undefined
[23]	39	5.79	Uveal Melanomas	Brachytherapy	10.26	Undefined
[24]	239	4.00	Uveal Melanomas	Brachytherapy	5.44	Undefined
[25]	52	3.92	Uveal Melanomas	Brachytherapy	38.46	Imaging
[26]	143	3.16	Uveal Melanomas	Brachytherapy	32.87	Imaging
[27]	50	5.20	Uveal Melanomas	Brachytherapy	0.00	Undefined
[28]	37	3.00	Uveal Melanomas	Brachytherapy	10.81	Undefined
[29]	47	3.91	Uveal Melanomas	Brachytherapy	51.00	Imaging
[30]	72	4.50	Uveal Melanomas	Brachytherapy	20.83	Imaging
[31]	95	10.00	Uveal Melanomas	Brachytherapy	24.21	Undefined
[32]	94	3.83	Uveal Melanomas	Brachytherapy	62.77	Imaging
[33]	36	3.00	Uveal Melanomas	Brachytherapy	0.00	Imaging
[34]	95	14.98	Uveal Melanomas	Brachytherapy	24.21	Undefined
[35]	400	4.26	Uveal Melanomas	Brachytherapy	5.75	Imaging
[36]	650	5.00	Uveal Melanomas	Brachytherapy	27.38	Imaging
[37]	134	5.00	Retinoblastomas	Brachytherapy	20.90	Imaging
[38]	31	1.80	Uveal Melanomas	Brachytherapy	16.13	Undefined
[39]	21	3.60	Uveal Melanomas	Hypofractionated External Beam RT (PT)	9.52	Imaging
[40]	96	3.50	Uveal Melanomas	Brachytherapy	45.83	Imaging
[41]	136	5.17	Uveal Melanomas	Brachytherapy	3.68	Mixture
[42]	354	10.00	Uveal Melanomas	Brachytherapy	22.03	Undefined
[43]	578	5.8	Uveal Melanomas	Hypofractionated External Beam RT	0.87	Undefined
[44]	94	2.80	Uveal Melanomas	Brachytherapy	9.57	Imaging
[45]	59	4.00	Uveal Melanomas	Hypofractionated External Beam Fractionated RT	32.20	Clinical
[46]	93	6.50	Uveal Melanomas	Brachytherapy	51.61	Imaging
[47]	93	10.00	Uveal Melanomas	Brachytherapy	81.72	Mixture
[48]	39	1.16	Uveal Melanomas	Brachytherapy	35.90	Clinical
[49]	53	1.30	Uveal Melanomas	Brachytherapy	13.21	Clinical

The definitions and diagnostic criteria for RION varied (**Table S1**). The diagnosis relied on optic disc changes (edema, pallor, hemorrhage, atrophy), with some studies requiring visual acuity loss as a criterion and others focusing on optic nerve appearance alone. Imaging modalities such as optical coherence tomography (OCT), fluorescein angiography, and fundus photography were inconsistently applied.

Several factors were commonly associated with an increased risk of RION (**Error! Reference source not found.**). These included tumor proximity to the posterior pole [16], [26], [40], [44], [48]; higher radiation doses, and doses to the optic nerve exceeding 50 Gy [17], [19], [24], [29], [36], [47]; larger tumor dimensions, such as height, thickness, or plaque size [22], [26], [31]; and the presence of retinal detachment prior to treatment. Demographic and clinical risk factors such as White or Caucasian race 43, age over 45 years 46, diabetes [36], [46], and elevated intraocular pressure 43 at diagnosis were also identified.

Two studies were identified as outliers on the basis of interquartile range (IQR) analysis [32], [47]. These studies reported RION rates of 81.72% and 62.77%, significantly exceeding rates reported in the other studies. Both investigations focused specifically on juxtapapillary choroidal melanomas, located at or in close proximity to the optic nerve and often involving the peripapillary region or extending to the optic disc.

Excluding these outliers, the meta-analysis of the remaining 34 studies yielded a pooled RION incidence of 17.03% (95% CI: 12.06% to 23.49%) (Fig. 2). Sensitivity analyses indicated that the study by Rudoler et al. (1997), which focused on choroidal metastases, disproportionately influenced the pooled estimate, warranting cautious interpretation. Leave-one-out analysis revealed pooled incidence estimates ranging from 16.32% to 17.77% among the other studies (Fig. S1). Significant heterogeneity was observed (I^2^ = 92.0%, *p* < 0.01). Meta-regression revealed that studies with undefined RION criteria reported significantly lower incidence rates (*p* = 0.02) (**Table S3**).

**Fig. 2:**
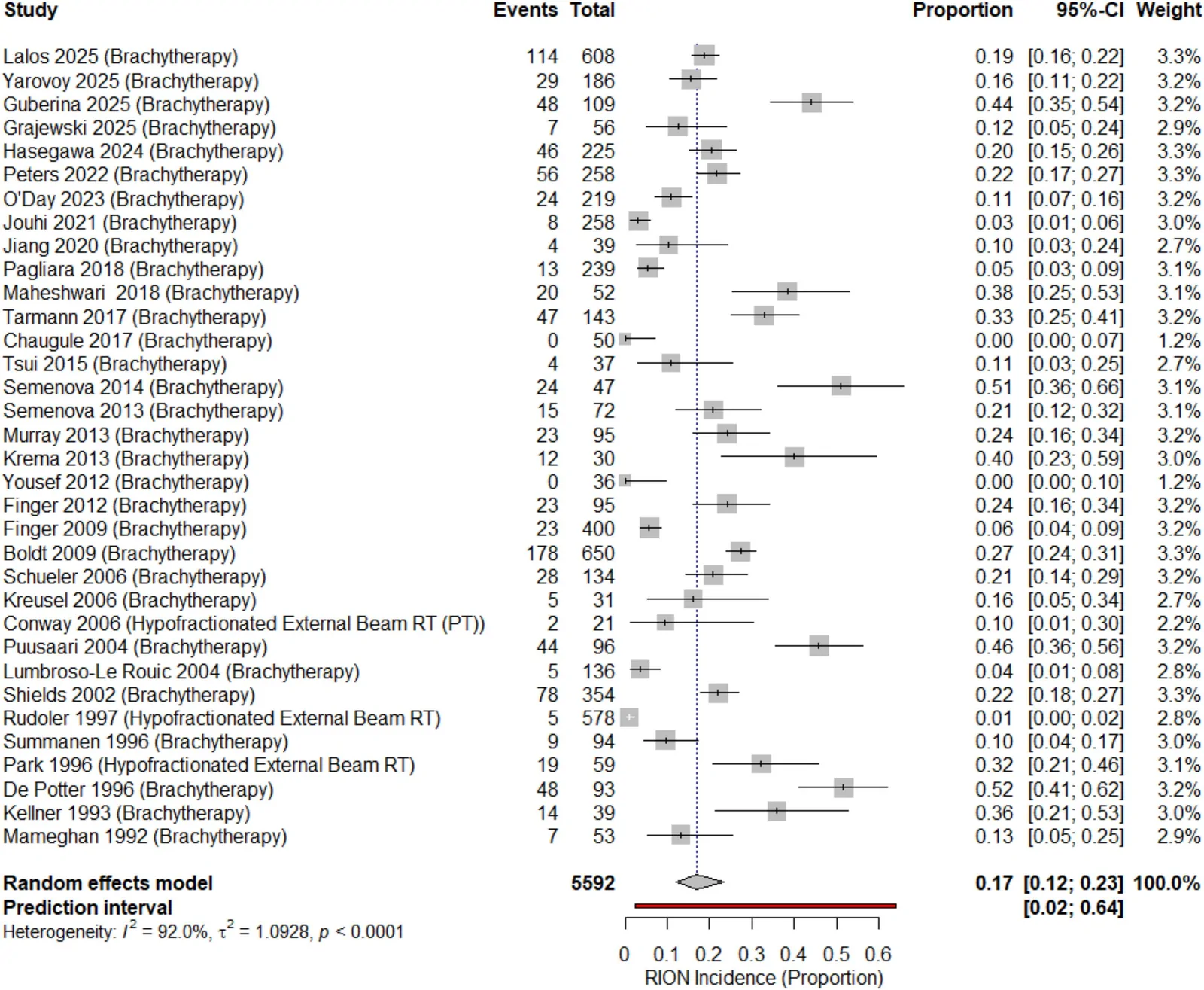
Meta-analysis of the incidence of RION in intraocular tumors.

### 3.2 RION in CNS/head-and-neck extraocular tumors

Radiotherapy was either conventionally fractionated or hypofractionated. Hypofractionated radiotherapy is increasingly employed in the management of small intracranial tumors but its use in head and neck cancers remains limited. This section begins by analyzing the full dataset, followed by stratified subpopulation analyses of conventionally fractionated or hypofractionated radiotherapy studies. Of 67 studies, 39 utilized conventionally fractionated and 28 hypofractionated regimens. The pooled incidence of RION was lower in the hypofractionated group (2.05%) than in the conventionally fractionated group (4.68%, *p* = 0.01). However, hypofractionated studies had a shorter median follow-up (3.40 years vs. 5.07 years, *p* < 0.001).

Both CNS and head-and-neck tumors were treated with conventionally fractionated radiotherapy, whereas hypofractionated studies were limited to CNS tumors. The incidence of RION was significantly greater in patients with head-and-neck cancer (HNC) than in those with CNS tumors.

An interquartile range statistic identified four studies as statistical outliers, each reporting RION rates exceeding 10%. Three studies used more precise RION diagnostic techniques and reported higher RION rates: (1) Astradsson et al. (2014a), which conducted a prospective study with detailed posttreatment follow-up, including magnetic resonance imaging (MRI), visual acuity, and visual field testing 50; (2) Ozkaya Akagunduz et al. (2017); and (3) Leber et al. (1998a) used visual evoked potentials to detect RION in addition to standard clinical assessments [2], [51]. In contrast, Katz et al. (2002) reported a head-and-neck cohort treated with two-dimensional conventional radiotherapy 52. These diagnostic techniques are commonly employed in the management of ocular tumors but have so far rarely been used in CNS/head-and-neck tumors.

After these outliers were excluded, the pooled RION incidence across the remaining studies was 3.18% (95% CI: 2.25–4.03), with significant heterogeneity (I^2^ = 70.1%, *p* < 0.001) (Fig. 3). The dataset revealed a wide range of RION incidences, with several studies reporting absence of RION [53], [54], [55] and others, particularly in head-and-neck cohorts, reporting high incidences in the order of 10% [56], [57], [58], [59]. The meta-regression analysis revealed that tumor type and fractionation influenced the incidence of RION in extraocular tumors. Head-and-neck tumors were significantly associated with a greater incidence of RION (estimate = 0.7185, *p* = 0.0076) than CNS tumors. In addition, hypofractionation was associated with a trend toward a lower incidence of RION (estimate = −0.5186, *p* = 0.0716). This apparent effect of hypofractionation may be confounded by tumor type since no head-and-neck patients underwent hypofractionated radiotherapy (**Table S4, Table S45**).

**Fig. 3:**
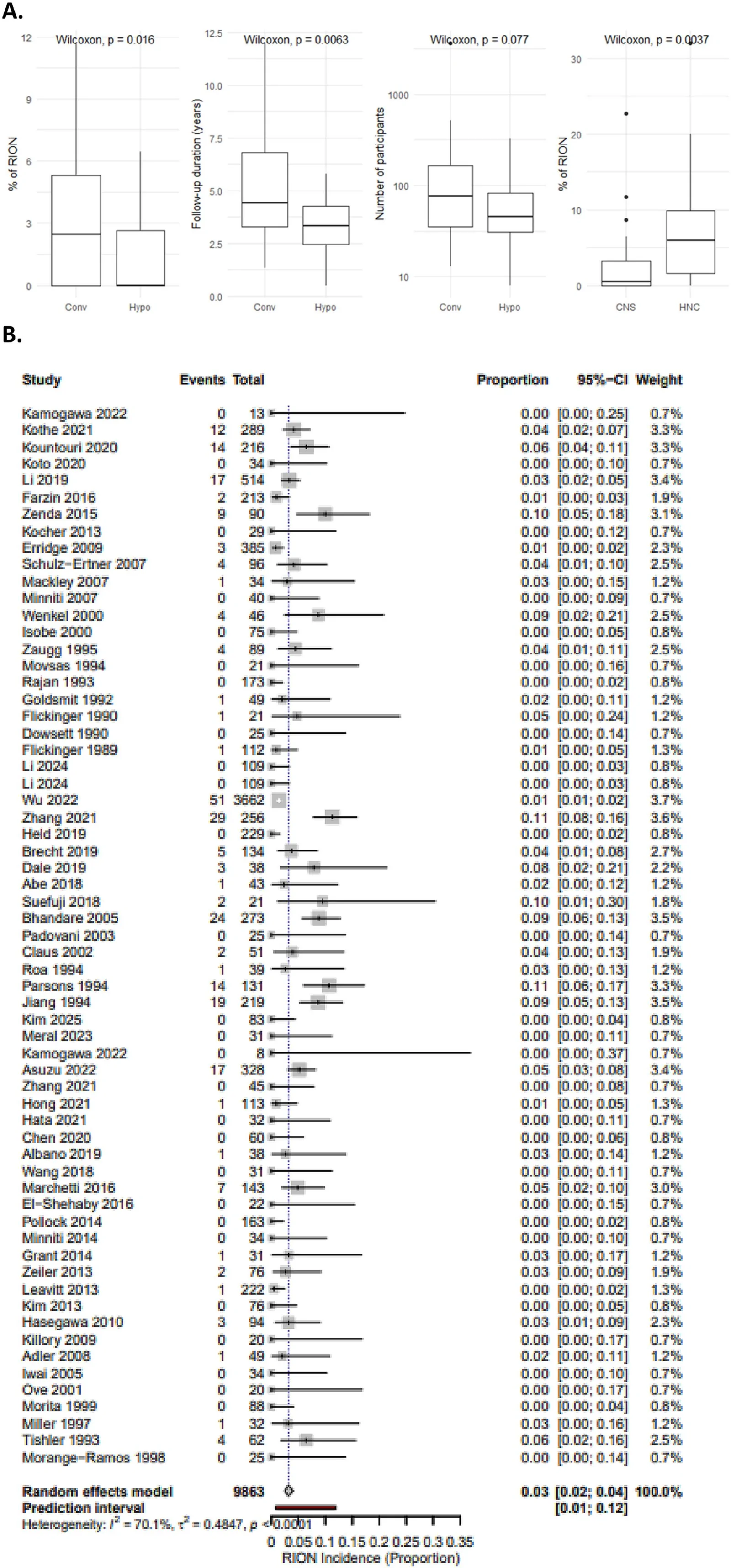
Meta-analysis of the incidence of RION in extraocular tumors.

#### 3.2.1 Subpopulation analysis: conventionally fractionated radiotherapy

A total of 39 studies related to conventionally fractionated radiotherapy published between 1989 and 2024 were included in this analysis, encompassing a broad spectrum of tumor types, radiotherapy modalities, and follow-up durations (Table 2). The sample sizes ranged from 20 to 3662 patients, with follow-up periods varying from 1.33 to 12 years. These studies focused primarily on nasopharyngeal carcinoma, skull-base tumors, chordomas, and chiasmatic tumors, such as pituitary adenomas, craniopharyngiomas, and cavernous sinus meningiomas, as well as head-and-neck cancers.

**Table 2:** Radiation-induced optic neuropathy incidence in series on extraocular tumors following conventionally fractionated radiotherapy.

First Authors	Study size	Follow-up period	Tumor Type	Radiotherapy type	RION	RION Definition	Dose	Dose per Fraction
[60]	109	1.72	HNC	CIRT	0.00	Clinical	83.7	2.7
[60]	109	1.72	HNC	IMRT	0.00	Clinical	68.2	2.2
[53]	13	2.72	CNS	IMRT	0.00	Clinical	54	1.8
[61]	3662	5.98	HNC	IMRT	1.39	Clinical	71	2.22
[62]	289	5.60	CNS	PBT	4.15	Clinical	71.2	1.9
[56]	256	7.25	HNC	IMRT	11.33	Clinical	71	2.22
[63]	216	5.00	CNS	PBT	6.48	Combined clinical and imaging	74	1.9
[55]	34	9.00	CNS	CIRT	0.00	Clinical	60.8	3.8
[64]	514	4.80	CNS	PBT	3.31	Combined clinical and imaging	70	1.89
[65]	229	3.90	HNC	CIRT	0.00	Clinical	51	3
[66]	134	4.29	HNC	IMRT	3.73	Clinical	60.5	2
[67]	38	3.92	HNC	CIRT	7.89	Clinical	69.6	4.4
[68]	43	2.50	HNC	CIRT	2.33	Clinical	63.9	3.1
[59]	21	3.25	HNC	CIRT	9.52	Clinical	63.1	3.26
[51]	20	3.42	HNC	3DCRT	20.00	Combined clinical and imaging	70	2.1
[69]	213	6.25	CNS	3DCRT	0.94	Clinical	54	2
[58]	90	4.79	HNC	PBT	10.00	Clinical	64.7	2.72
[50]	55	6.80	CNS	3DCRT	11.7	Clinical	54	1.9
[70]	29	3.75	CNS	FSRT	0.00	Clinical	52.2	1.8
[71]	385	9.10	CNS	3DCRT	0.78	Clinical	45	1.8
[72]	96	2.58	CNS	CIRT	4.17	Clinical	60	3
[73]	34	3.54	CNS	IMRT	2.94	Clinical	45.9	1.7
[74]	40	9.00	CNS	3DCRT	0.00	Clinical	45.75	1.8
[75]	273	10.00	HNC	2DCRT	8.79	Clinical	61	1.15
[76]	25	2.08	HNC	3DCRT	0.00	Clinical	63	2
[52]	78	5.00	HNC	2DCRT	32.05	Clinical	68	1.15
[77]	51	2.67	HNC	3DCRT	3.92	Clinical	65	1.9
[78]	46	4.42	CNS	PBT	8.70	Clinical	59	1.8
[79]	75	8.17	CNS	3DCRT	0.00	Clinical	50	2
[80]	89	10.00	CNS	3DCRT	4.49	Clinical	43	2
[81]	21	4.00	CNS	3DCRT	0.00	Clinical	50	1.9
[57]	39	4.50	HNC	3DCRT	2.56	Clinical	68.4	1.9
[1]	131	3.00	HNC	3DCRT	10.69	Clinical	54	1.2
[82]	219	1.92	HNC	2DCRT	8.68	Clinical	57.1	2
[83]	173	12.00	CNS	2DCRT	0.00	Clinical	56	1.52
[84]	49	3.83	CNS	2DCRT	2.04	Clinical	51.62	2.31
[85]	21	7.30	CNS	2DCRT	4.76	Clinical	60	1.83
[86]	25	4.42	CNS	2DCRT	0.00	Clinical	46	2
[87]	112	1.33	CNS	2DCRT	0.89	Clinical	44.17	1.84

Legend: HNC: head and neck cancer; BOS: skull-based tumor; CNS: central nervous system tumor; IMRT: intensity-modulated radiation therapy; CIRT: carbon ion radiation therapy; PBT: proton beam therapy; 3DCRT: three-dimensional conformal radiation therapy; 2DCRT: two-dimensional conventional radiation therapy; FSRT: fractionated stereotactic radiotherapy.

The reported incidence of RION varied widely across studies, ranging from 0% to 32.05%. The highest incidences were typically observed in studies employing older two-dimensional radiotherapy (2DCRT), such as Katz (2002), who reported a 32.05% incidence. In contrast, several more recent studies using advanced techniques such as IMRT, proton therapy, or carbon ion radiotherapy reported no RION cases. The definitions of RION were not uniform (**Table S6**). Some studies adopted standardized criteria, such as the Common Terminology Criteria for Adverse Events (CTCAE) [58], [59], [65], [67], [69], [88]. Others applied the RTOG/EORTC criteria 72, symptomatic evaluations of visual acuity and visual field [1], [52], [57], [61], [66], [80], [82], [83], [86], or ophthalmologic assessments such as visual evoked potentials, optical coherence tomography, or magnetic resonance imaging (MRI) findings [51], [60], [63]. Several studies did not define RION at all.

In terms of associated risk factors (**Table S7**), the radiation dose to the optic apparatus, especially the maximum dose (Dmax) to the optic nerve or chiasm, was the most consistently reported predictor [51], [57], [61], [67], [78]. The total radiation dose was also associated with RION in several studies [1], [73], [75], [87]. Additional factors linked to increased risk included older age, female sex, hypertension, multiple surgeries before radiotherapy, and tumor proximity to the optic pathway, in two proton therapy studies at the same institution [62], [63]. However, some studies did not identify any specific predictors or contributing factors.

The meta-analysis of 38 studies (excluding 1 outlier study from IQR analysis 52) yielded a pooled RION incidence of 3.79% (95% CI: 2.78% to 5.16%) (Fig. 4). The analysis revealed substantial heterogeneity (I2 = 79.3%, *p* value <0.001). Leave-one-out sensitivity analysis revealed that the pooled incidence remained stable, ranging from 2.87% to 3.68% (Fig. S2). Meta-regression revealed that the total dose administered to the tumor was significantly associated with the incidence of RION (estimate = 0.0457, *p* = 0.043; **Table S8**). The same model did not identify a statistically significant association between recruitment period, used as a proxy for treatment era, and RION incidence (estimate = −0.0208 per year, 95% CI: −0.0515 to 0.0098, *p* = 0.183; Table S8), indicating no statistically significant temporal trend within this subgroup. The meta-analysis of data stratified by tumor type revealed that for CNS tumors, the pooled RION incidence was 3.27% (95% CI: 2.34% to 4.56%) (Q = 27.42, *p* = 0.15), suggesting low heterogeneity. In contrast, head-and-neck cancer patients had a higher pooled incidence of 4.79% (95% CI: 2.75% to 8.22%), with significant heterogeneity observed (Q = 148.42, *p* < 0.001) (Fig. 5).

**Fig. 4:**
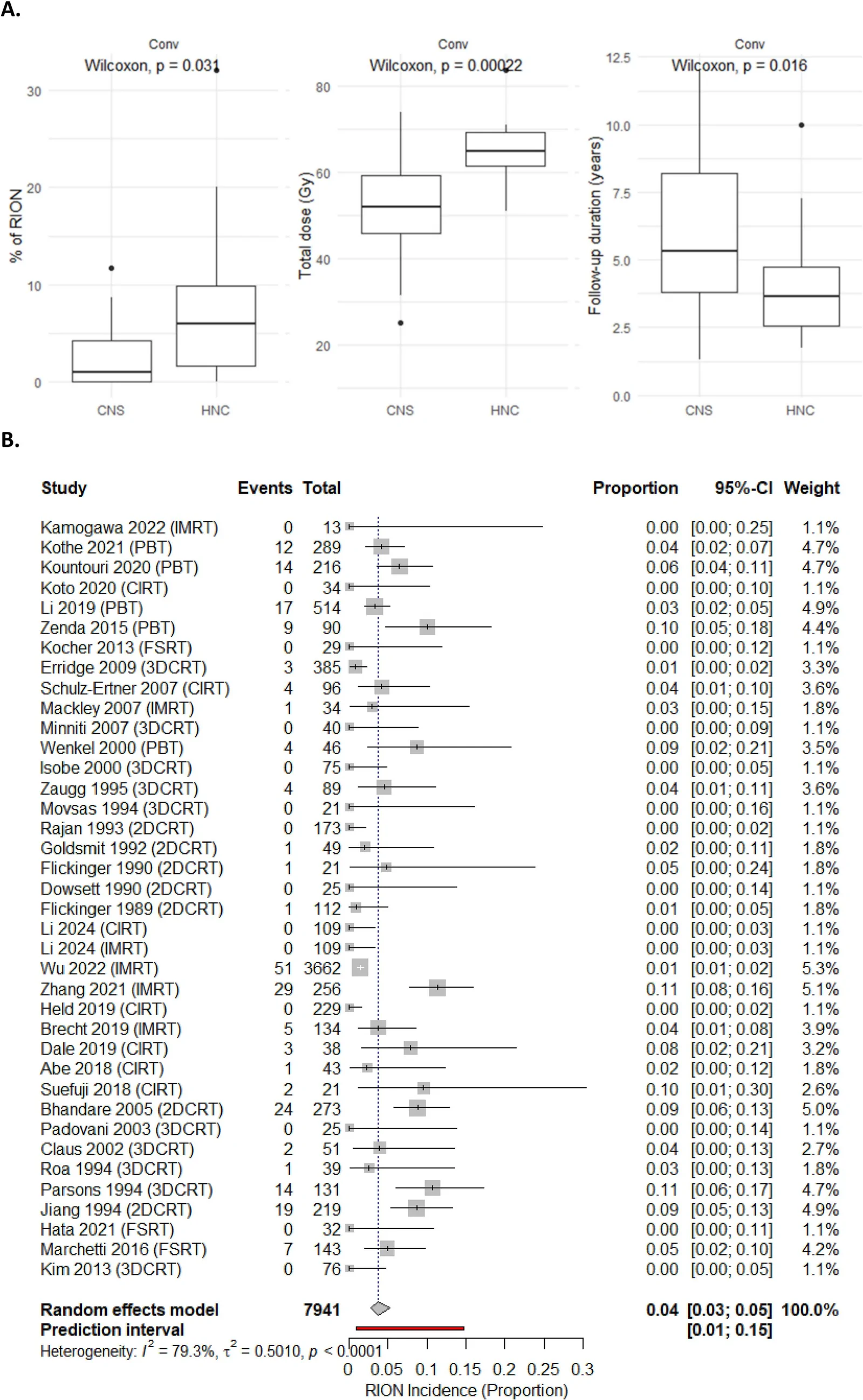
Meta-analysis of the incidence of RION in extraocular tumors treated with conventionally fractionated radiotherapy.

**Fig. 5:**
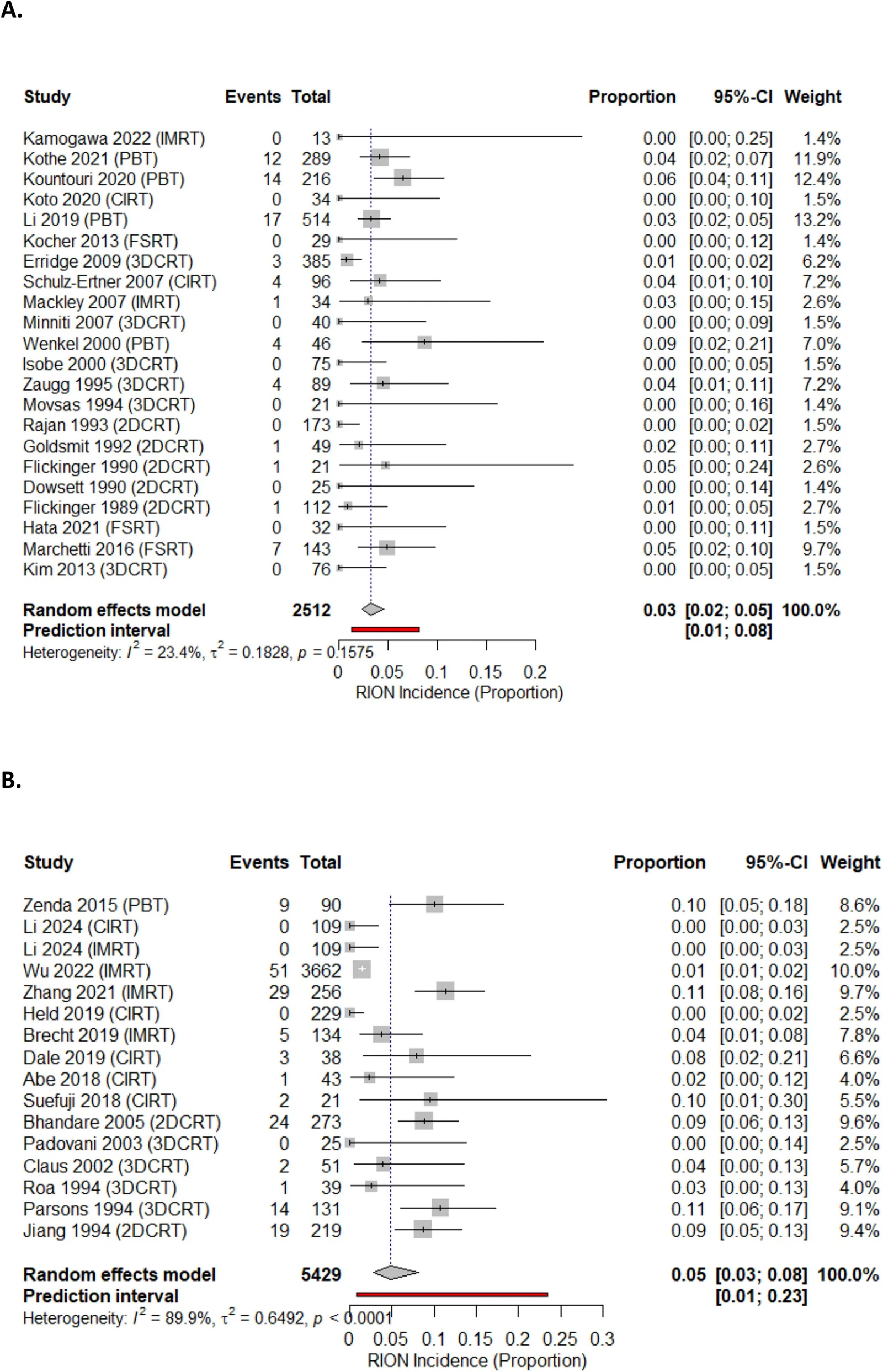
Meta-analysis of the incidence of RION in extraocular tumors treated with conventionally fractionated radiotherapy stratified by tumor type: (A) CNS; (B) HNC.

#### 3.2.2 Subpopulation analysis: hypofractionated radiotherapy

Twenty-eight studies related to hypofractionated radiotherapy, including diverse tumor types such as pituitary adenomas, meningiomas, craniopharyngiomas, and other skull base tumors, were included, published between 1993 and 2025 (Table 3). The number of patients per study varied widely, ranging from 20 to 85. The follow-up periods ranged from approximately 1.1 to over 10 years. A variety of radiotherapy modalities, including gamma knife radiosurgery (GKRS) and fractionated stereotactic radiotherapy (FSRT), were used across these studies. These techniques generally resulted in low or null RION incidence. Nonetheless, follow-up periods were shorter than those in conventionally fractionated studies, possibly resulting in underreporting of long-term toxicities.

**Table 3:** Studies on extraocular tumors and the incidence of radiation-induced optic neuropathy following hypofractionated external beam radiotherapy.

Studies	Study Size	Follow-up (years)	Tumor Type	Radiotherapy Type	RION (%)	RION Diagnostic Method
[54]	83	4.70	Parachiasmatic	GKRS	0.00	Clinical
[89]	31	5.17	Parachiasmatic	GKRS	0.00	Undefined
[53]	21	2.72	Parachiasmatic	GKRS	0.00	Undefined
[90]	328	4.67	BOS (meningioma)	GKRS	5.18	Clinical
[91]	45	4.28	Parachiasmatic	GKRS	0.00	Undefined
[92]	113	4.08	BOS (meningioma)	GKRS	0.88	Clinical
[93]	60	5.80	Paraoptic tumor	FSRT	0.00	Clinical
[94]	143	3.67	Parachiasmatic	FSRT	4.90	Clinical
[95]	22	3.58	Paraoptic tumor	GKRS	0.00	Clinical
[96]	163	2.67	Parachiasmatic	GKRS	0.00	Imaging
[97]	34	1.08	BOS (metastasis)	FSRT	0.00	Clinical
[98]	31	3.35	Parachiasmatic	GKRS	3.23	Clinical
[99]	76	2.73	Parachiasmatic	GKRS	2.63	Clinical
[100]	222	10.25	BOS (Benign tumor)	GKRS	0.45	Clinical
[70]	29	3.75	Paraoptic tumor	FSRT	0.00	Clinical
[101]	76	6.80	Parachiasmatic	FSRT	0.00	Undefined
[102]	49	4.08	Paraoptic tumor	GKRS	2.04	Clinical
[103]	34	4.98	Parachiasmatic (Pituitary adenoma)	GKRS	0.00	Clinical
[104]	88	2.67	BOS (meningioma)	GKRS	0.00	Clinical
[2]	66	3.33	BOS (Benign tumor)	GKRS	22.73	Combined clinical and imaging
[105]	32	2.30	BOS (Benign tumor)	GKRS	3.13	Clinical
[106]	62	1.58	Parachiasmatic	GKRS	6.45	Clinical
[107]	38	3.72	Parachiasmatic	GKRS	2.63	Clinical
[108]	31	2.50	Parachiasmatic	FSRT	0.00	Clinical
[109]	20	2.44	Parachiasmatic	FSRT	0.00	Clinical
[110]	20	2.00	Parachiasmatic	GKRS	0.00	Clinical
[111]	25	1.67	Parachiasmatic	GKRS	0.00	Undefined

Legend: BOS: skull-based tumor; SRS: stereotactic radiosurgery, GKRS: gamma knife radiosurgery, FSRT: fractionated stereotactic radiotherapy, SRS/SRT (6 MV LINAC): stereotactic radiosurgery/stereotactic radiotherapy using a 6 Megavolt linear accelerator. Parachiasmatic tumors include pituitary adenomas, craniopharyngiomas, and cavernous sinus meningiomas.

The diagnosis of RION varied considerably across studies (Table **S9**). Some used standardized grading systems such as CTCAE [92], [101], [105], [108], whereas others relied on clinical findings, including deterioration of visual acuity and visual field defects [50], [70], [71], [90], [93], [94], [98], [99], [100], [102], [103], [104], [106], [107], [109], [110], [112], [113], visual evoked potential changes ^2^, or subjective symptoms of vision loss not attributed to tumor recurrence 107. Several studies did not clearly define RION.

Most studies did not identify any specific predictive factors for RION (**Table S10**). However, among studies that did, the most consistently reported factor was the dose to the optic nerve or optic apparatus [2], [19], [94], [98], [106]. Other studies highlighted preexisting visual field impairment 50 and lower Karnofsky performance status prior to radiotherapy 90 as contributing risk factors.

The pooled proportion of RION, calculated via a random-effects model, was 0.58% (95% CI: 0.09% to 1.36%) (Fig. 6). Heterogeneity among the studies was significant, with an I^2^ statistic of 39.8% (*p* value = 0.02). Leave-one-out sensitivity analysis revealed that the pooled RION incidence ranged from 2.12% to 2.61% (**Fig. S3**). The highest incidence of RION was reported in Tishler et al. (1993), with a rate of 6.45%. The elevated rate in this series is likely attributable to inadequate dose constraints, as reported in this study, 24% of patients who received more than 0.8 Gy to any part of the optic apparatus developed visual complications.

**Fig. 6:**
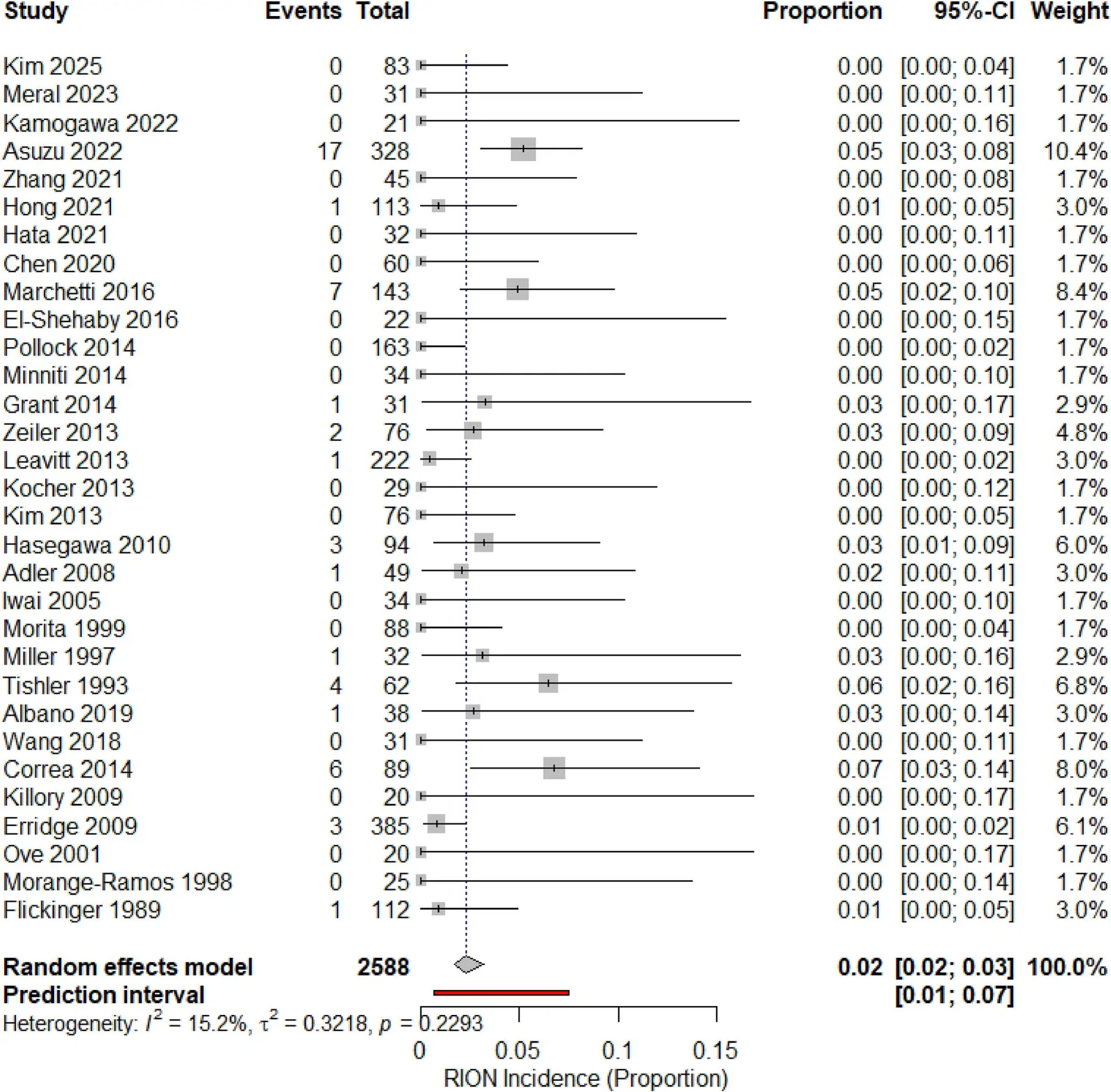
Meta-analysis of the incidence of RION in extraocular tumors treated with hypofractionated radiotherapy.

#### 3.2.3 Subpopulation analysis: CNS tumor studies of conventionally fractionated or hypofractionated radiotherapy

This subpopulation analysis included 47 studies of CNS tumors. The pooled incidence of RION was estimated at 2.77% (95% CI: 2.19% to 3.50%) via a random effects model, with a prediction interval of 1.05% to 7.09%. Between-study heterogeneity was low to moderate, with an I^2^ of 13%, and the test for heterogeneity was not statistically significant (Q = 52.77, *p* = 0.22). In mixed-effects meta-regression models, fractionation type (hypofractionation, *p* = 0.54), dose per fraction (*p* = 0.77), and EQ.D2 (*p* = 0.36) were not significantly associated with RION incidence across CNS tumor studies.

### 3.3 Temporal trends in RION incidence

Meta-regressions did not identify a statistically significant association between publication year and RION incidence in intraocular tumors (OR per decade 0.87, 95% CI 0.56–1.35, *p* = 0.51), extraocular tumors treated with conventionally fractionated radiotherapy (OR 0.94, 95% CI 0.67–1.34, *p* = 0.74) or CNS tumors (OR 0.90, 95% CI 0.68–1.19, *p* = 0.46) (Table S11, Fig. S4). Results were consistent when the midpoint of the recruitment period replaced publication year, after exclusion of IQR-defined outliers, and after adjustment for follow-up duration, RION definition, tumor type and dose (all *p* ≥ 0.05). In hypofractionated series, a nominal decline (OR per decade 0.63, 95% CI 0.42–0.96, *p* = 0.03) was driven by a single early radiosurgery series with a high reported incidence ^2^ and disappeared after its exclusion (OR 0.88, 95% CI 0.61–1.26, *p* = 0.47). In head-and-neck cohorts, a borderline decline was observed with recruitment period (OR per decade 0.67, 95% CI 0.45–0.99, *p* = 0.044), which was not robust to outlier exclusion or to adjustment for follow-up (*p* = 0.065 and *p* = 0.053, respectively). Pooled incidences stratified by publication era (<2000, 2000–2009 and ≥ 2010) did not differ significantly in any subgroup, e.g., 22.9%, 16.1% and 19.6% in intraocular tumors (*p* = 0.81) and 4.0%, 4.8% and 4.1% in conventionally fractionated extraocular tumors (*p* = 0.95) (Table S12).

Because RION ascertainment changed over time, an exploratory model of extraocular tumors was additionally adjusted for the use of imaging-based RION definitions and for tumor type. Imaging-based ascertainment was associated with a higher reported incidence (OR 3.16, 95% CI 1.45–6.92, *p* = 0.005), and a modest decline over time emerged (OR per decade 0.78, 95% CI 0.61–0.98, *p* = 0.035). This trend was not robust, being attenuated after exclusion of outliers (OR 0.85, 95% CI 0.68–1.06, *p* = 0.14) and in a permutation test (*p* = 0.06).

### 3.4 Sensitivity analyses and risk of bias

When all studies were retained, the pooled RION incidence was 19.18% (95% CI 13.48–26.55%; I^2^ = 94%) in intraocular tumors, 4.18% (2.91–5.97%; I^2^ = 86%) in conventionally fractionated and 2.62% (1.62–4.22%; I^2^ = 59%) in hypofractionated extraocular series, compared with 17.03%, 3.99% and 2.58%, respectively, after exclusion of IQR-defined outliers (Table S13). Outlier exclusion therefore mainly reduced heterogeneity rather than modifying point estimates. Prediction intervals were wide (intraocular 1.9–74.9%; all extraocular 0.6–18.8%), underscoring the limited generalizability of single pooled values. A GLMM, which avoids continuity corrections in zero-event studies, yielded lower estimates in extraocular tumors (e.g., CNS 1.33% vs 2.88%), reflecting the large proportion of studies without reported RION events.

Follow-up duration was not associated with RION incidence in any subgroup (all *p* ≥ 0.36). Among CNS tumors, hypofractionated and conventionally fractionated series did not differ after adjustment for follow-up (OR 1.31, 95% CI 0.68–2.54, *p* = 0.41) or when restricted to studies with a median follow-up of at least 4 years (2.9% vs 3.4%, *p* = 0.61). As surrogates for clinically relevant optic exposure, restricting conventionally fractionated extraocular series to prescribed doses of ≥50 Gy, ≥54 Gy or ≥ 60 Gy yielded pooled incidences of 4.8%, 5.5% and 5.4%, respectively, and no temporal trend was observed among perichiasmatic tumors (OR per decade 0.98, *p* = 0.91).

Methodological quality was moderate to high (NOS 6–9/9). The comparability criterion was most frequently unmet, in 20 of 35 intraocular and 9 of 28 hypofractionated studies. The NOS score was not associated with RION incidence in intraocular or conventionally fractionated studies (both *p* = 0.70), whereas higher-quality hypofractionated studies reported lower incidences (OR per NOS point 0.46, *p* = 0.044). After adjustment for NOS score, publication year remained unassociated with incidence in intraocular and conventionally fractionated studies (*p* = 0.40 and *p* = 0.84).

## 4 Discussion

RION remains a challenging complication, particularly given the lack of effective treatment options once vision loss occurs. While NTCP models have been instrumental in advancing our understanding of dose–response relationships and guiding treatment planning, they require that RION estimates are accurate and risks for RION well identified. Our meta-analysis demonstrates considerable variability in RION incidence by tumor location, radiotherapy modality and radiation dose. The analysis also showed differences in duration of follow-up, as well as definitions of RION and quality of the assessments. Reporting biases can be suspected in many studies, depending on whether RION is self-reported by the patients or using endpoints such as visual acuity that are indeed not specific to RION but rather indicative of retinopathy and multifactorial factors. Systematic examinations performed in all patients via standardized and quantitative exams have more potential but involve more staff and are more costly, with a lack of health economics studies for evaluating systematic assessments. Loss to follow-up due to competing events, such as death or tumor progression, is often not accounted for in the statistics, which represents a substantial source of reporting bias. Population selection is quite critical to assess RION, and sampling biases should be stated explicitly by describing the study populations in detail. Risk of bias was assessed using the Newcastle–Ottawa Scale. Most included studies were of moderate-to-high methodological quality, but the comparability criterion was frequently unmet because few studies adjusted for optic dose, tumor location or follow-up; study quality did not modify the temporal findings. For example, intraocular tumors treated with brachytherapy or proton therapy presented lower RION rates than extraocular tumors treated with conventionally fractionated or hypofractionated radiotherapy did, but the dose distributions were dramatically different. In intraocular tumors, the retina is significantly exposed, while the nerve is usually spared except in the first mm of the optic nerve head, which is composed of the ganglion cell layer of the retina. In contrast, in extraocular tumors, the optic nerve head is minimally exposed, but the conic or intracanal or the brain portions of the optic nerves can be exposed to significant doses approaching their threshold tolerance doses. In addition, the calculated dose to the optic apparatus was not always reported or explicitly stated. For accurate risk assessment, using the biologically effective dose (BED) to the optic structures or the dose expressed as EQ.D2 terminology is important for study intercomparison. Where possible, future research should specify the optic apparatus dose and calculation methods, acknowledging potential discrepancies between target prescription and true optic dose. Ultimately, the risk of RION is strongly related to the dose received by the optic structures [8], [54]. Most contemporary treatment protocols aim to minimize optic dose, but practice variation exists. Outlier cases, where optic structures are deliberately irradiated to high doses, for example, >70 Gy in skull base chordomas without optic sparing, can disproportionately increase reported incidence. Such cases should be identified and contextualized, as they do not reflect standard vision-preserving practices. This variability highlights the importance of considering tumor-specific and treatment-specific factors when assessing RION risk. The observed heterogeneity also highlights the challenges in drawing generalizable conclusions from pooled data, necessitating robust statistical methodologies and careful study design in future research. The pooled estimates should therefore be interpreted as descriptive summaries of heterogeneous literature rather than universal incidence values. In particular, the high heterogeneity among intraocular studies indicates substantial variation in tumor geometry, treatment technique, dose, RION definition and follow-up; subgroup and sensitivity analyses are therefore more informative than the overall pooled estimate. Recent prospective proton-therapy work has extended NTCP modeling beyond irreversible grade 4 toxicity used visual-field deficits and dosiomics to predict grade ≥ 2 RION, identifying baseline visual deficit and chiasmal dose-volume exposure as important predictors, supporting the need to combine functional and dosimetric information rather than rely on dose constraints alone 114.

Other underexplored factors include sex and associated treatments. Sex-specific incidence could not be analyzed because sex-stratified RION events were inconsistently reported across studies. The concurrent or sequential use of systemic agents can alter radiosensitivity. For instance, temozolomide in CNS tumors or concurrent chemotherapy in head and neck cancers may increase optic nerve susceptibility to radiation injury [115], [116]. Many studies provided incomplete treatment details, limiting our ability to analyze their impact on RION incidence. However, for ocular tumors (uveal melanomas), compared with FSRT, proton therapy appears to minimize the dose to the optic disk and the risk of RION 117, with the potential for multibeam proton therapy to further improve the dose to the optic nerve compared with single-beam proton therapy 118. In addition, brachytherapy is often unsuitable for juxtapapillary/macular tumors, due to presence of the optic nerve on the sclera side where the plaque is placed. This limitation may lead to either tumor undercoverage or RION. In CNS/head-and-neck tumors, choosing to hypofractionate the radiotherapy and the choice of the particle are dependent on many parameters, including tumor histology, tumor size, tumor distance to the optic structures among others. In particular, although crude RION incidence was lower in hypofractionated series, these studies were restricted to CNS tumors and had shorter follow-up. The difference should therefore not be interpreted as evidence of lower RION risk with hypofractionation. In CNS-only meta-regression, fractionation type, dose per fraction and EQ.D2 were not significantly associated with RION incidence. Therefore, conclusions on the effects of fractionation should be interpreted with caution. From a clinical standpoint, improved radiotherapy conformality does not necessarily translate into a proportional decline in RION incidence. For tumors abutting the optic apparatus, treatment planning remains constrained by optic-nerve and chiasmal tolerance, and adequate target coverage may require doses approaching these limits. Technological advancements may therefore help minimize unnecessary exposure to optic structures in many radiotherapy types, but other physical and radiobiological quantities, functional tissue assessment by advanced MRI sequences, and individual radiosensitivity, may be needed for accurate and robust predictive modeling of RION. In addition, improved tumor control and survival may increase the number of patients observed long enough to develop late toxicity. Consequently, temporal trends in RION reflect not only technological evolution but also tumor geometry, dose selection, survival, follow-up, and ascertainment. Finally, machine learning algorithms trained on large datasets have the potential to refine dose constraints and predict late toxicity more accurately, facilitating individualized care. Indeed, Bayesian networks has already been used to model conditional relationships among clinical, ophthalmological, and treatment variables; baseline visual-field deficit, hypertension, and hypercholesterolemia emerged as direct predictors, underscoring the potential contribution of vascular comorbidity and pre-existing optic-pathway impairment to individualized risk assessment 119.

Another challenge in current research on RION is the lack of standardized definitions, which complicates the comparison of findings across studies. Temporal comparisons are additionally confounded by evolution in RION ascertainment. Earlier studies often relied on symptomatic visual loss or non-standardized clinical criteria, whereas more recent studies increasingly incorporate formal visual-field testing, electrophysiology, MRI and OCT. Greater diagnostic sensitivity in contemporary cohorts may increase detection of subclinical or earlier events and could therefore mask a true reduction in severe clinically manifest RION over time. Consistent with this, in an exploratory model of extraocular tumors, imaging-based RION definitions were associated with an approximately threefold higher reported incidence, and adjustment for ascertainment revealed a modest but non-robust decline over time. The variability in follow-up durations and inconsistent reporting of outcomes further hinder efforts to establish clear conclusions regarding RION risk and predictors. Follow-up duration is particularly important for RION because it is a delayed toxicity that may occur months to years after irradiation. Consequently, studies with shorter observation periods may systematically underestimate incidence. This consideration is relevant to the present comparison because hypofractionated CNS series had a shorter median follow-up than conventionally fractionated series (3.4 vs 5.1 years), limiting direct interpretation of differences in crude incidence. Reported latencies range from a few months to more than a decade, with median onset approximately 16–30 months after irradiation [15], [17], [57]; most included series therefore had a median follow-up exceeding the median latency, but not its upper range. Nevertheless, follow-up duration was not significantly associated with incidence in meta-regression, and the comparison between hypo- and conventionally fractionated CNS series remained non-significant after adjustment for follow-up. Moreover, most NTCP models are based on retrospective data, which may not fully capture the dynamic nature of treatment responses or long-term outcomes. A further limitation is the inability to apply a uniform minimum optic-structure dose threshold across studies. Many reports did not provide the number of patients receiving doses above a predefined level, precluding a consistent sensitivity analysis restricted to clinically relevant exposure. As a pragmatic surrogate, restricting conventionally fractionated series to prescribed doses of 50–60 Gy or more yielded slightly higher pooled incidences (4.8–5.5%), and no temporal trend was observed among perichiasmatic tumors. Future studies should report dose-stratified denominators to facilitate such analyses. Finally, a key limitation is the insufficient exploration of dose–response relationships, especially in the low- and high-dose ranges where data scarcity introduces significant uncertainty. Consistent with the importance of adequate observation time, a mixed-effects analysis of longitudinal visual fields after proton therapy found progressive, nonlinear sensitivity loss, with age, target volume, and chiasmal V40 improving prediction and simulated grade ≥ 2 RION risk increasing from 4.6% at 2 years to 28.3% at 5 years 120. Multimodal prospective assessment after proton therapy further showed that visual-field mean deviation and pattern standard deviation identified RION more effectively than visual acuity alone, with functional and electrophysiological abnormalities appearing earlier than retinal nerve-fiber-layer thinning; however, the modest cross-validated performance of combined models supports cautious interpretation and external validation 121. In addition, archetypal analysis of visual fields in a proton-therapy cohort identified interpretable patterns of loss and associations with chiasmal dose, illustrating how longitudinal functional phenotyping may complement incidence-based meta-analysis and help characterize clinically meaningful visual outcomes 122.

To address these gaps and validate predictive models, prospective studies that collect comprehensive dosimetric, imaging, and clinical data are needed. In particular, for contemporary NTCP development, standardized RION definitions should be coupled with harmonized optic-structure dose-volume metrics and adequate long-term follow-up. Future models should evaluate fractionation, tumor–optic geometry, longitudinal imaging biomarkers and patient-level susceptibility in addition to dose, while distinguishing prescription dose from the actual dose delivered to the optic nerves and chiasm. Standardized protocols for patient assessments are crucial but are demanding in terms of staffing and cost. For this reason, populations at greater risk, such as those with tumors located near the optic nerves, are often included in studies to increase the likelihood of obtaining meaningful outcomes. Unfortunately, the absence of predictive biomarkers to select patients at risk of RION limits the precision of these models. Future research should aim to develop a consensus definition of RION and establish standardized protocols for risk evaluation. Such efforts should also explore the comparative value of different ophthalmologic examinations, such as visual field testing, optical coherence tomography, and electrophysiologic studies, in identifying early changes and refining patient risk stratification. Establishing these standards would enhance the comparability of studies, improve the predictive accuracy of models, and ultimately inform evidence-based strategies to minimize RION.

Three practical messages emerge from this analysis. First, long-term ophthalmological surveillance remains important for patients with paraoptic tumors, i.e., intraocular tumors close to the optic disc and extraocular tumors abutting the optic nerves or chiasm, because RION is a delayed toxicity that may occur several years after irradiation and was observed across all treatment eras and techniques. Second, modern radiotherapy technology alone may not eliminate RION risk near critical optic structures: when the target abuts the optic apparatus, adequate tumor coverage may still require doses approaching tolerance, and no significant decline in RION incidence was observed over three decades. Third, individualized risk assessment and NTCP modeling remain essential; optic-structure dosimetry should be interpreted together with tumor–optic geometry, fractionation, pre-existing visual impairment, ocular and systemic comorbidities and concurrent treatments, both for treatment planning and for informing patients about the risk of visual loss.

To improve comparability across studies and to enable pooled NTCP modeling, we recommend that future studies of RION report, at a minimum: (i) optic-structure dose metrics, including the maximum dose to each optic nerve and to the chiasm with the volume definition used, the corresponding EQ.D2 or BED with the assumed α/β ratio (and RBE for particle therapy), the dose per fraction, and the number of patients exceeding predefined dose thresholds; (ii) diagnostic criteria for RION, including an explicit definition, the grading system and its version, the ophthalmological examinations performed (visual acuity, visual field, fundoscopy, OCT, visual evoked potentials, MRI) and their schedule, and the criteria used to exclude tumor progression or other causes of visual loss; (iii) follow-up duration, including the median and range of ophthalmological follow-up, the number of patients at risk over time, the latency to RION onset, and the handling of competing events such as death or tumor progression, ideally with actuarial or competing-risk estimates in addition to crude proportions; and (iv) patient-level dosimetric data, i.e., individual or dose-binned optic-structure doses linked to RION outcomes and baseline visual status, shared as supplementary data or in public repositories to allow external validation of dose–response models.

## 5 Conclusion

No statistically significant temporal trend in RION incidence was detected between 1994 and 2023 despite improvements in radiotherapy techniques. RION is rare but remains a debilitating condition across all radiotherapy techniques, for which no curative treatment exists. However, using high-resolution ophthalmological imaging-based monitoring, RION can now be detected earlier and better characterized, providing clues for more effective treatments in the future. Standardized definitions of RION, consistent reporting of outcomes, and long-term follow-up will be critical in refining NTCP models and ensuring optimal care for patients at risk of this disabling visual complication.

## CRediT authorship contribution statement

**Juliette Thariat:** Writing – review & editing, Writing – original draft, Visualization, Validation, Supervision, Software, Resources, Project administration, Methodology, Investigation, Funding acquisition, Data curation, Conceptualization. **Thao-Nguyen Pham:** Writing – review & editing, Writing – original draft, Visualization, Validation, Methodology, Formal analysis, Data curation, Conceptualization. **Thomas Sochacki:** Writing – review & editing, Writing – original draft, Visualization, Data curation. **Florent de Vathaire:** Writing – review & editing, Writing – original draft, Visualization, Data curation. **Helen A. Shih:** Writing – review & editing, Writing – original draft, Visualization. **Jean-Claude Quintyn:** Writing – review & editing, Writing – original draft, Visualization.

## Funding

The funders (PMRT ERDF-FSE 2014–2020 no 18P03532/18 E01765; SEQ-RTH22 No 2023–017) of the study had no role in study design, data collection, data analysis, data interpretation, or writing of the report.

## Declaration of competing interest

The authors declare that they have no known competing financial interests or personal relationships that could have appeared to influence the work reported in this paper.

## Appendix A Supplementary data

Supplementary material: Table S1. RION diagnostic methods reported in studies of intraocular tumors.
