# Supplementary material for: Temporal trends in radiation-induced optic neuropathy: a meta-analysis
Source: Clin Transl Radiat Oncol. 2026 Oct 18;62:101327. doi: 10.1016/j.ctro.2026.101327 (PMC13742046; doi:10.1016/j.ctro.2026.101327)
Supplement: Supplementary file 1 [file mmc1.docx]

# Supplementary

**Abbreviations used in the Supplementary Material:** BCVA, best-corrected visual acuity; CI, confidence interval; CNS, central nervous system; CTCAE, Common Terminology Criteria for Adverse Events; FSRT, fractionated stereotactic radiotherapy; GKRS, gamma knife radiosurgery; GLMM, generalized linear mixed model; IQR, interquartile range; MRI, magnetic resonance imaging; OCT, optical coherence tomography; OR, odds ratio; REML, restricted maximum likelihood; RION, radiation-induced optic neuropathy; RT, radiotherapy; RTOG/EORTC, Radiation Therapy Oncology Group/European Organisation for Research and Treatment of Cancer; SE, standard error; SRS, stereotactic radiosurgery; VA, visual acuity; VECP/VEP, visual evoked cortical potential/visual evoked potential; VF, visual field.

Table S1. RION diagnostic methods reported in studies of intraocular tumors

| Studies | RION diagnostic methods |
| --- | --- |
| ^86^ | Undefined |
| ^19^ | Undefined |
| ^25^ | Disc edema, vaso-obliteration, hemorrhage, cotton wool spots, disc pallor. |
| ^90^ | Visual acuity, clinical examination, sonography, and fundus photography |
| ^24^ | Optic neuropathy defined by disc edema or pallor, excluding other causes. |
| ^91^ | Comprehensive ophthalmic examination and VA |
| ^92^ | New optic nerve swelling or pallor observed. |
| ^27^ | Undefined |
| ^93^ | Undefined |
| ^23^ | Undefined |
| ^94^ | Fundus photography, ultrasonography, and OCT. |
| ^18^ | Optic neuropathy defined as partial or complete optic nerve atrophy with or without optic disc hemorrhage. |
| ^95^ | Undefined |
| ^96^ | Undefined |
| ^22^ | Undefined |
| ^97^ | Optic nerve atrophy, characterized by end-stage postirradiation changes. |
| ^26^ | Undefined |
| ^30^ | Optic disc swelling with peripapillary hemorrhages, exudates, or disc pallor |
| ^98^ | Fundus photography, fluorescein angiography, and optical coherence tomography for screening |
| ^99^ | Undefined |
| ^100^ | Fluorescein angiography to evaluate tumor circulation, leakage, radiation retinopathy, and optic neuropathy. |
| ^21^ | Optic disc edema, hemorrhage, and/or pallor |
| ^101^ | Optic disc edema with or without local hemorrhage or lipid exudates |
| ^102^ | Undefined |
| ^103^ | Partial or complete optic disc pallor |
| ^17^ | Edema, peripapillary hemorrhages, microinfarcts or optic atrophy |
| ^104^ | Visual acuity and systematic search for complications. Optic neuropathy confirmed by fluorescein angiography. |
| ^105^ | Undefined |
| ^28^ | Undefined |
| ^16^ | Ophthalmologic examination. Development of anterior ischemic optic neuropathy caused by vascular occlusion, seen as edema, hemorrhages, microinfarcts, and narrowing of optic nerve |
| ^106^ | Visual field perimetry, enlargement of absolute scotoma of less than or equal to 30° compared to pretreatment visual field |
| ^29^ | Peripapillary exudation and hemorrhage, optic disc swelling, or optic disc neovascularization |
| ^20^ | Visual acuity and optic disc injury |
| ^15^ | Pathological VECPs |
| ^107^ | Visual acuity |

Table S2. Factors associated with RION reported in intraocular tumors

| Studies | RION associated factors |
| --- | --- |
| ^86^ | Unspecified |
| ^19^ | Posterior pole, tumor thickness > 2.7 mm, plaque diameter > 14 mm |
| ^25^ | Radiation dose |
| ^90^ | Unspecified |
| ^24^ | White race, Posterior pole (<6 mm of optic nerve), radiation dose |
| ^91^ | Retinal detachment before treatment |
| ^92^ | Unspecified |
| ^27^ | Plaque size |
| ^93^ | Unspecified |
| ^23^ | Optic nerve dose (> 50 Gy), Posterior pole (< 4 mm of optic disk) |
| ^94^ | Unspecified |
| ^18^ | Posterior pole and largest basal tumor diameter |
| ^95^ | Unspecified |
| ^96^ | Unspecified |
| ^22^ | Radiation dose to the tumor |
| ^97^ | Unspecified |
| ^26^ | Tumor height |
| ^30^ | Greater rate of RION in Iodine-125 brachytherapy compared to Stereotactic Radiation Therapy |
| ^98^ | Unspecified |
| ^99^ | Unspecified |
| ^100^ | Unspecified |
| ^21^ | Diabetes, Posterior pole, optic nerve head dose |
| ^101^ | Retinal detachment before brachytherapy |
| ^102^ | Unspecified |
| ^103^ | Unspecified |
| ^17^ | Posterior pole |
| ^104^ | Unspecified |
| ^105^ | Unspecified |
| ^28^ | Caucasian race, increased intraocular pressure at diagnosis (>21 mmHg) |
| ^16^ | Posterior pole |
| ^106^ | Unspecified |
| ^29^ | Age (>45 years), diabetes, mushroom-shaped tumor, nasal tumor location |
| ^20^ | Optic nerve dose (≥50 Gy) |
| ^15^ | Posterior pole |
| ^107^ | Unspecified |

Table S3. Meta-regression model results for RION incidence in intraocular tumors

|  | Estimate | SE | p value | CI low | CI high |
| --- | --- | --- | --- | --- | --- |
| Intercept | -1.2644 | 0.5961 | 0.0339* | -2.4329 | -0.0960 |
| RION definition: Imaging | -0.2484 | 0.6399 | 0.6979 | -1.5027 | 1.0058 |
| RION definition: Mixture | -1.2695 | 0.8304 | 0.1263 | -2.8970 | 0.3580 |
| RION definition: Undefined | -1.6316 | 0.7429 | 0.0281* | -3.0875 | -0.1756 |
| Follow-up period (years) | 0.0974 | 0.0712 | 0.1712 | -0.0421 | 0.2368 |

Table S4. Meta-regression model results for RION incidence in extraocular tumors by tumor type

|  | Estimate | SE | p value | CI low | CI high |
| --- | --- | --- | --- | --- | --- |
| Intercept | -3.6390 | 0.1613 | <.0001 | -3.9552 | -3.3229 |
| Tumor type: Head and Neck | 0.7185 | 0.2693 | 0.0076 | 0.1907 | 1.2464 |

Table S5. Meta-regression model results for RION incidence in extraocular tumors by fractionation type

|  | Estimate | SE | p value | CI low | CI high |
| --- | --- | --- | --- | --- | --- |
| Intercept | -3.2391 | 0.1632 | <.0001 | -3.5589 | -2.9193 |
| Hypofractionation | -0.5186 | 0.2878 | 0.0716 | -1.0827 | 0.0456 |

Table S6. RION diagnostic methods reported in studies of extraocular tumors following conventionally fractionated radiotherapy

| Studies | RION diagnostic methods |
| --- | --- |
| ^53^ | Not defined |
| ^51^ | Visual field loss or BCVA deterioration by one grade, excluding tumor-related or comorbid causes |
| ^37^ | Irreversible optic/chiasmal dysfunction without other causes |
| ^58^ | CTCAE v5.0, confirmed by MRI |
| ^52^ | Progressive VA loss with visual field defect + abnormal VEP or optic disc pallor |
| ^36^ | Not defined |
| ^108^ | Visual loss and/or MRI enhancement ≥6 months post-RT, excluding recurrence or other causes |
| ^43^ | CTCAE v5.0 |
| ^50^ | Blindness, hemianopsia, or ≥2-point VA loss not explained by tumor or surgery |
| ^42^ | CTCAE |
| ^109^ | Optic nerve disorder |
| ^40^ | CTCAE |
| ^32^ | Multimodal eye/optic nerve testing including VA, VF, contrast sensitivity, VEP, OCT |
| ^41^ | CTCAE |
| ^39^ | CTCAE |
| ^45^ | RTOG/EORTC |
| ^57^ | Visual deficit |
| Minniti | Not defined |
| ^56^ | Not defined |
| ^111^ | Not defined |
| ^33^ | Visual deficit |
| ^112^ | Not defined |
| ^54^ | Ophthalmologic toxicity |
| ^113^ | Not defined |
| ^49^ | Long-term visual complication |
| ^114^ | Ophthalmologic toxicity |
| ^38^ | Visual acuity ≤ 20/100 due to optic nerve injury |
| ^1^ | Visual acuity < 20/100 |
| ^48^ | Visual field deficit or visual acuity loss |
| ^47^ | Visual field deficit or visual acuity loss |
| ^115^ | Not defined |
| ^116^ | Not defined |
| ^46^ | Visual field deficit or visual acuity loss |
| ^55^ | Not defined |

Table S7. Factors associated with RION reported in extraocular tumors following conventionally fractionated radiotherapy

| Studies | RION associated factors |
| --- | --- |
| ^53^ | No mention/No factor found |
| ^51^ | Dmax to optic nerve/chiasma |
| ^37^ | Dmax to optic nerve/chiasma |
| ^58^ | Age, hypertension, ≥3 surgeries prior to RT |
| ^52^ | Age, hypertension, tumor abutting optic apparatus |
| ^36^ | No mention/No factor found |
| ^108^ | Higher dose, older age, female sex |
| ^43^ | No mention/No factor found |
| ^50^ | No mention/No factor found |
| ^42^ | Dose to optic nerve |
| ^109^ | No mention/No factor found |
| ^40^ | No mention/No factor found |
| ^32^ | Dose–volume effects on optic nerves |
| ^41^ | No mention/No factor found |
| ^39^ | No mention/No factor found |
| ^45^ | No mention/No factor found |
| ^57^ | Total dose > 46 Gy |
| (Minniti et al. 2007) | No mention/No factor found |
| ^56^ | Total dose, patient age |
| ^111^ | No mention/No factor found |
| ^33^ | No mention/No factor found |
| ^112^ | No mention/No factor found |
| ^54^ | Dose to optic nerve |
| ^113^ | No mention/No factor found |
| ^49^ | No mention/No factor found |
| ^114^ | No mention/No factor found |
| ^38^ | Dose to optic nerve, age |
| ^1^ | Total dose |
| ^48^ | No mention/No factor found |
| ^47^ | No mention/No factor found |
| ^115^ | No mention/No factor found |
| ^116^ | Total dose |
| ^46^ | No mention/No factor found |
| ^55^ | No mention |

Table S8. Meta-regression model results for RION incidence in extraocular tumors following conventionally fractionated radiotherapy

|  | Estimate | SE | p value | CI low | CI high |
| --- | --- | --- | --- | --- | --- |
| Intercept | 35.6952 | 30.4948 | 0.2418 | -24.0734 | 95.4639 |
| Recruitment period | -0.0208 | 0.0157 | 0.1830 | -0.0515 | 0.0098 |
| Total dose | 0.0457 | 0.0226 | 0.0432 | 0.0014 | 0.0899 |

Table S9. RION diagnostic methods reported in studies of extraocular tumors following hypofractionated radiotherapy

| Studies | RION diagnostic methods |
| --- | --- |
| ^35^ | CTCAE; no optic neuropathy or visual decline post SRS |
| ^117^ | Non-defined |
| ^34^ | Non-defined |
| ^79^ | Ophthalmic visual field examination |
| ^118^ | Non-defined |
| ^62^ | CTCAE grade 1 |
| ^78^ | Visual acuity, Goldman visual field exams |
| ^77^ | Vision loss defined by >2 Snellen lines or 2 dB VF drop, clinical complaints |
| ^75^ | Visual field and acuity assessment |
| ^119^ | Visual field examination |
| ^120^ | Pre/post SRS ophthalmological exams |
| ^121^ | Visual acuity and field tests |
| ^74^ | Visual field deficits |
| ^31^ | RION defined by VA/VF symptoms |
| ^31^ | Visual symptoms in absence of other lesions/tumor growth |
| ^73^ | Visual field defect |
| ^72^ | Loss of >2 VA lines and/or ≥2 dB VF deviation |
| ^71^ | Visual acuity loss >0.1 or new VF defect |
| ^60^ | Non-defined |
| ^70^ | Visual acuity and field |
| ^67^ | Visual loss unrelated to tumor |
| ^66^ | Visual loss unrelated to tumor |
| ^64^ | Visual loss unrelated to tumor |
| ^2^ | VEP latency/amplitude change, VF defects, VA loss |
| ^59^ | CTCAE |
| ^63^ | Visual complications |
| ^76^ | Visual decline without tumor recurrence |
| ^61^ | CTCAE |
| ^61^ | CTCAE |
| ^69^ | Visual field testing |
| ^68^ | Visual deterioration |
| ^65^ | Visual field perimetry |
| ^122^ | Non-defined |

Table S10. Factors associated with RION reported in extraocular tumors following hypofractionated radiotherapy

| Studies |  |
| --- | --- |
| ^35^ | No factor found |
| ^117^ | No factor found |
| ^34^ | No factor found |
| ^79^ | Karnofsky Performance Status pre-SRS |
| ^118^ | No factor found |
| ^62^ | No factor found |
| ^78^ | No factor found |
| ^77^ | No factor found |
| ^75^ | Optic nerve dose |
| ^119^ | No factor found |
| ^120^ | No factor found |
| ^121^ | No factor found |
| ^74^ | Optic nerve dose |
| ^31^ | Pre-FSRT visual field impairment |
| ^31^ | Pre-FSRT visual field impairment |
| ^73^ | No factor found |
| ^72^ | No factor found |
| ^71^ | No factor found |
| ^60^ | No factor found |
| ^70^ | Optic apparatus dose |
| ^67^ | No factor found |
| ^66^ | No factor found |
| ^64^ | No factor found |
| ^2^ | Optic nerve dose |
| ^59^ | No factor found |
| ^63^ | Optic nerve dose |
| ^76^ | No factor found |
| ^61^ | No factor found |
| ^61^ | No factor found |
| ^69^ | No factor found |
| ^68^ | No factor found |
| ^65^ | No factor found |
| ^122^ | No factor found |

Figure S1. Sensitivity analysis of RION reported in intraocular tumors

Figure S2. Sensitivity analysis of RION reported in extraocular tumors following conventionally fractionated radiotherapy

Figure S3. Sensitivity analysis of RION reported in extraocular tumors following hypofractionated radiotherapy
Table S11. Meta-regression of RION incidence on publication year and recruitment period, by subgroup (odds ratio per decade, 95% CI, p value)

| **Subgroup (k)** | **Publication year, all studies** | **Publication year, excluding outliers** | **Recruitment midpoint, all studies** |
| --- | --- | --- | --- |
| **Unadjusted models** | | | |
| Intraocular (36) | 0.87 (0.56–1.35), p = 0.51 | 0.99 (0.66–1.48), p = 0.95 | 0.84 (0.55–1.28), p = 0.40 |
| Intraocular, brachytherapy only (32) | 0.75 (0.50–1.12), p = 0.16 | 0.88 (0.59–1.29), p = 0.49 | 0.72 (0.49–1.05), p = 0.09 |
| Extraocular, conventionally fractionated (39) | 0.94 (0.67–1.34), p = 0.74 | 0.99 (0.72–1.37), p = 0.96 | 0.91 (0.69–1.20), p = 0.48 |
| Extraocular, hypofractionated (28) | 0.63 (0.42–0.96), p = 0.03 | 0.88 (0.61–1.26), p = 0.47 | 0.65 (0.41–1.05), p = 0.08 |
| All extraocular (67) | 0.83 (0.64–1.08), p = 0.16 | 0.89 (0.70–1.11), p = 0.29 | 0.82 (0.66–1.03), p = 0.08 |
| CNS, conventionally fractionated and hypofractionated (49) | 0.90 (0.68–1.19), p = 0.46 | 1.02 (0.80–1.29), p = 0.88 | 0.99 (0.76–1.28), p = 0.93 |
| Head and neck (18) | 0.66 (0.37–1.18), p = 0.15 | 0.71 (0.43–1.17), p = 0.16 | 0.67 (0.45–0.99), p = 0.04 |
| **Adjusted models** | | | |
| Intraocular; follow-up, RION definition (36) | 1.07 (0.66–1.73), p = 0.79 | 1.28 (0.83–1.99), p = 0.25 | 1.00 (0.64–1.55), p = 1.00 |
| Conventionally fractionated; tumor type, follow-up, dose (39) | 0.71 (0.47–1.06), p = 0.09 | 0.79 (0.53–1.18), p = 0.24 | 0.74 (0.55–1.00), p = 0.05 |
| Hypofractionated; follow-up (28) | 0.57 (0.36–0.92), p = 0.02 | 0.84 (0.54–1.29), p = 0.41 | 0.61 (0.35–1.08), p = 0.09 |
| CNS; fractionation, follow-up (49) | 0.92 (0.70–1.23), p = 0.58 | 1.05 (0.82–1.33), p = 0.70 | 1.02 (0.77–1.35), p = 0.87 |
| Head and neck; follow-up (18) | 0.63 (0.35–1.13), p = 0.11 | 0.66 (0.40–1.10), p = 0.10 | 0.67 (0.45–1.01), p = 0.05 |
| All extraocular; tumor type, fractionation, follow-up (67) | 0.81 (0.63–1.05), p = 0.11 | 0.89 (0.71–1.11), p = 0.29 | 0.83 (0.66–1.03), p = 0.09 |
| All extraocular; tumor type, imaging-based definition* (67) | 0.78 (0.61–0.98), p = 0.035 | 0.85 (0.68–1.06), p = 0.14 | 0.81 (0.66–0.98), p = 0.03 |

Random-effects meta-regression (REML, Knapp–Hartung) on logit-transformed proportions; OR < 1 indicates decreasing incidence over time. IQR-defined outliers: intraocular, Lommatzsch 1994 and Krema 2013 (stereotactic arm); conventionally fractionated, Katz 2002; hypofractionated, Leber 1998; all extraocular, Katz 2002, Leber 1998, Astradsson 2014 and Ozkaya Akagunduz 2017. *Exploratory model: imaging-based definition OR 3.16 (95% CI 1.45–6.92), p = 0.005; permutation p for publication year = 0.06. The recruitment period was unavailable for one intraocular and one hypofractionated study.

Table S12. Pooled RION incidence by publication era

| **Subgroup** | **<2000: k; % (95% CI)** | **2000–2009: k; % (95% CI)** | **≥2010: k; % (95% CI)** | **p between eras (all / excl. outliers)** |
| --- | --- | --- | --- | --- |
| Intraocular | 7; 22.9 (6.5–56.1) | 8; 16.1 (8.6–28.1) | 21; 19.6 (13.1–28.3) | 0.81 / 0.94 |
| Extraocular, conventionally fractionated | 10; 4.0 (2.0–7.7) | 9; 4.8 (1.9–11.2) | 20; 4.1 (2.5–6.5) | 0.95 / 0.96 |
| Extraocular, hypofractionated | 5; 5.3 (1.5–17.5) | 4; 2.0 (0.6–6.7) | 19; 2.3 (1.5–3.8) | 0.47 / 0.71 |
| All extraocular | 15; 4.4 (2.4–8.0) | 13; 4.1 (1.9–8.4) | 39; 3.2 (2.2–4.5) | 0.62 / 0.62 |
| CNS | 12; 3.3 (1.5–7.2) | 9; 2.6 (1.2–5.3) | 28; 3.0 (2.1–4.2) | 0.89 / 0.96 |
| Head and neck | 3; 9.1 (6.6–12.5) | 4; 9.5 (2.8–28.0) | 11; 4.2 (2.0–8.8) | 0.16 / 0.07 |

Random-effects inverse-variance model (REML) on logit-transformed proportions; all studies included. p values for subgroup differences are given with all studies and after exclusion of IQR-defined outliers.

Table S13. Pooled RION incidence with and without IQR-defined outliers, and with an alternative pooling model

| **Subgroup (k all / k excl.)** | **All studies: % (95% CI); I²** | **Excluding outliers: % (95% CI); I²** | **GLMM, all studies: % (95% CI)** | **95% prediction interval, all studies** |
| --- | --- | --- | --- | --- |
| Intraocular (36/34) | 19.18 (13.48–26.55); 94% | 17.03 (12.37–22.98); 92% | 17.84 (12.20–25.35) | 1.9–74.9% |
| Extraocular, conventionally fractionated (39/38) | 4.18 (2.91–5.97); 86% | 3.99 (2.86–5.52); 81% | 2.60 (1.57–4.28) | 0.6–23.0% |
| Extraocular, hypofractionated (28/27) | 2.62 (1.62–4.22); 59% | 2.58 (1.75–3.79); 0% | 0.87 (0.34–2.23) | 0.4–14.1% |
| All extraocular (67/63) | 3.55 (2.65–4.73); 81% | 3.18 (2.45–4.11); 70% | 1.77 (1.11–2.81) | 0.6–18.8% |
| CNS (49/47) | 2.88 (2.09–3.96); 54% | 2.77 (2.11–3.62); 13% | 1.33 (0.77–2.29) | 0.6–12.1% |
| Head and neck (18/16) | 5.79 (3.39–9.73); 93% | 4.79 (2.94–7.70); 90% | 3.95 (1.92–7.92) | 0.7–36.4% |

Random-effects inverse-variance model (REML) on logit-transformed proportions with Wald-type 95% CI. GLMM: binomial-normal generalized linear mixed model without continuity correction. Prediction intervals were derived from the Hartung–Knapp model including all studies.

Figure S4. Meta-regression of RION incidence on publication year in (A) intraocular tumors and in extraocular tumors treated with (B) conventionally fractionated and (C) hypofractionated radiotherapy. Bubble size is proportional to study weight; black line, all studies (shaded band, 95% CI); orange line, excluding IQR-defined outliers (orange bubbles).

**Critical View section**

RION incidence varies according to tumor location, radiotherapy modality, and fractionation schemes, and no statistically significant temporal trend in incidence was detected between 1994 and 2023 despite major technological advances in radiation delivery. Clinical evidence based on our meta-analysis indicates that, although RION is strongly dose-dependent, its development is also modulated by additional factors, including pre-existing optic pathway damage (e.g., tumor compression) and ocular comorbidities (such as glaucoma or diabetes), as well as multimodal treatment approaches (e.g., surgery and chemotherapy). These observations highlight the limitations of purely dosimetric optimization strategies and underscore the need to shift toward biologically driven interventions. In particular, there is a critical need for therapeutic approaches, including drug development or repurposing, informed by a deeper mechanistic understanding of RION. High-resolution ophthalmological imaging offers a unique opportunity to dissect these mechanisms in vivo and to identify actionable targets for prevention and treatment.
